# Broaching Digital Twin to Predict Forces, Local Overloads, and Surface Topography Irregularities

**DOI:** 10.3390/ma17225471

**Published:** 2024-11-09

**Authors:** Gorka Ortiz-de-Zarate, Aitor Madariaga, Daniel Soler, Pedro J. Arrazola

**Affiliations:** Engineering Faculty, Mondragon Unibertsitatea, Loramendi 4, 20500 Arrasate-Mondragon, Spain; amadariaga@mondragon.edu (A.M.); dsoler@mondragon.edu (D.S.); pjarrazola@mondragon.edu (P.J.A.)

**Keywords:** digital twin, surface integrity, broaching, topography, residual stresses, aeronautic, Inconel 718, turbine disc, fir-tree, hybrid modelling

## Abstract

Broaching is a key manufacturing process that directly influences the surface integrity of critical components, impacting their functional performance in sectors such as aeronautics, automotive, and energy. Such components are subjected to severe conditions, including high thermomechanical loads, fatigue, and corrosion. For this reason, the development of predictive models is essential for determining the optimal tool design and machining conditions to ensure proper in-service performance. This study, therefore, presents a broaching digital twin based on hybrid modelling, which combines analytical, numerical, and empirical approaches to provide rapid and accurate predictions of the forces per tooth, local overloads, and surface topography irregularities. The digital twin was validated with a critical industrial case study involving fir-tree broaching of turbine discs made of forged and age-hardened Inconel 718. The accuracy of the digital twin was demonstrated by the results: the average error in force predictions was below 10%, and the model effectively identified the most critical teeth and zones prone to failure. It also predicted surface topography irregularities with an error of less than 15%. Interestingly, the relationship between surface topography irregularities and surface residual stress variations across the machined surface was observed experimentally for the first time.

## 1. Introduction

Broaching is one of the key machining processes in the manufacturing route of critical components for highly demanding sectors such as automotive (steering rack, wheel hubs, interchanges, differentials, etc.), aeronautics (fir-tree of turbine discs, compressor dovetail, etc.), or energy (heat exchangers in nuclear reactors, etc.) [1]. This process can generate very complex surfaces in a wide range of workpiece materials (e.g., aluminium, steel, stainless steel, hardened steel, titanium- and nickel-based alloys), with high dimensional quality (IT6/IT9) and surface integrity (smooth topography, minimal residual stresses, and no significant microstructural alterations) at a very competitive cost per part compared to other machining processes [1,2,3].

However, the broaching process has certain specificities that are not found in other machining operations. In addition to very wide chips, the long contact length between the workpiece and the tool, together with the simultaneous machining of several teeth, produces extremely high forces [1]. These can lead to premature breakage of the broaching tool and affect the surface integrity (topography, residual stresses, and microstructural damage) and geometrical tolerances of the machined component [4,5,6]. In particular, tooth entry and exit can locally generate abrupt surface topography height variations, also known as topography irregularities, as observed by Axinte when broaching the nickel-based alloy Inconel 718 [7]. In that work, the periodicity of these irregularities coincided with the pitch of the tool, and the author posited that they were a consequence of dynamic effects related to the stiffness of the machine tool–workpiece system. More recently, the authors of the present work also concluded that these irregularities are related to the stiffness of the system in combination with the magnitude and direction of the broaching forces [8].

Irregularities in the surface topography can lead to non-compliance with the strict geometric and dimensional tolerances established by the aforementioned sectors. A clear example is ring gears for electric vehicle transmissions, which are broached and require very close tolerances (quality 4–5 as specified by DIN 3961) to improve efficiency and NVH (Noise, Vibration, and Harshness) performance [9]. These irregularities can also negatively affect the fretting fatigue behaviour of assemblies, such as the joints between turbine blades and discs in an aeroengine [10]. Therefore, understanding and predicting the phenomena involved in the generation of these topography irregularities is of great scientific and industrial interest to ensure both the quality and the safe working life of the component.

In addition to topography, residual stresses are another critical aspect of surface integrity that impacts component performance [11,12,13,14,15]. Regardless of the machining process analysed, it has been consistently observed that compressive residual stresses are more beneficial than tensile stresses for fatigue life. For instance, several studies have examined the influence of residual stresses generated by turning on fatigue life using Inconel 718 [11,12,13] and TC17 titanium-based alloy [14,15] as workpiece materials. However, a limited number of authors have focused on residual stresses in broaching [4,5,16,17,18,19], and even fewer have explored their effect on fatigue life [16]. Most of the broaching studies analysing surface integrity have focused on Inconel 718 [5,6,7,8,16,17,18], the most widely used nickel-based alloy in aircraft engine components over the past 40 years [20] due to its high strength, excellent creep resistance, and fatigue life at elevated temperatures [21]. Despite the significant combined effect of residual stresses and surface topography on the performance of the component, no study has yet examined the variation in residual stresses across the broached surface or their relationship with surface topography irregularities.

Moreover, the manufacturing industry has a commitment to a continuous improvement objective of increasing the Material Removal Rate (MRR), so as to reduce costs, without compromising the surface integrity of the manufactured components. In the broaching process, only the cutting speed and lubrication can be modified. As a result, designing broaching tools to maximise the section of material that can be removed is one of the main factors in improving MRR [1,22,23]. To ensure optimised tool design and the proper selection of cutting conditions, it is crucial to consider the local loads acting on each section of the broaching teeth. Failure to consider these local loads can result in unexpected tool breakage or micro-cracking of the cutting edge (chipping).

Predictive models or digital twins can play a critical role in determining the total forces (for the design of clamping tools, part deformations, etc.) and the local mechanical loads during the broaching process [1,24]. Several authors have developed digital twins based on analytical models to optimise broaching tool design and improve productivity based on the total forces [22,23,24,25,26]. Mandrile et al. [25] developed a comprehensive study of the broaching process, in which they proposed a model capable of predicting the forces per tooth during fir-tree broaching on nickel-based alloy Udimet 720. A similar analytical approach was adopted by Bergs et al. [6], who selected the number of reworking/calibrated teeth (teeth without rise per tooth) by modelling the process-induced geometrical deviations when broaching half of a fir-tree of Inconel 718. However, in each of these works, the tool geometry is generally simplified or artificially generated without considering the geometrical peculiarities of the broaching tool such as the chipbreaker, heterogeneous material thickness along the tooth, etc.

Other authors have focused their efforts on modelling the broaching process using the Finite Element Method (FEM). Some studies have focused on fundamental outcomes (forces, chip morphology, and/or temperatures) [4,27,28,29,30], while others have tried to predict aspects of surface integrity [4,8,30]. Ortiz-de-Zarate et al. [8] developed a new methodology combining FEM simulation and experimental tests to predict topography in broaching steel, titanium, and nickel-based alloys. However, the computational cost of these simulations and the financial resources dedicated to the characterisation of the input parameters for the FEM models (flow stress, friction, ductile failure, etc.) are substantial [29,31,32,33].

In summary, no work has been found in the literature that proposes a digital twin that can predict the forces per tooth and the local loads for any broaching operation only by defining the geometry of the tool and the workpiece. Previous broaching studies have focused on optimising tool geometry by considering the total forces but have not examined the possible breakage of the cutting edge due to excessive local loads. Furthermore, no holistic model has been developed to determine the effect of broaching forces on critical surface integrity aspects such as topography irregularities. The occurrence of these topography irregularities in critical industrial operations also remains unexplored, as does their impact on other aspects of surface integrity, such as residual stresses, which directly influence the fatigue performance of the component.

Therefore, this paper presents a digital twin to predict forces, local loads, and topography irregularities in the manufacture of any complex shape geometry by broaching. Starting from a 3D geometry of the tool and workpiece, the digital twin extracts the information of the section of material to be machined for each tooth and predicts the total forces using a database of specific forces. The local load and critical teeth can then be determined from the thickness to be machined along the cutting edge. The calculation time is rapid (less than 5 min), making this model suitable for industrial applications to (i) optimise the broaching process and tool design, (ii) indicate possible tool wear during production, and (iii) identify non-conformities in surface topography if established threshold values are exceeded. The digital twin was validated with a critical industrial case study involving fir-tree broaching of turbine discs made of Inconel 718, in which its accuracy in predicting forces, local loads, and topography irregularities was demonstrated. The experimental investigation also examined the relationship between surface topography irregularities and surface residual stress variations of the broached fir-trees.

## 2. Materials and Methods

### 2.1. Digital Twin Development

**Figure 1:**
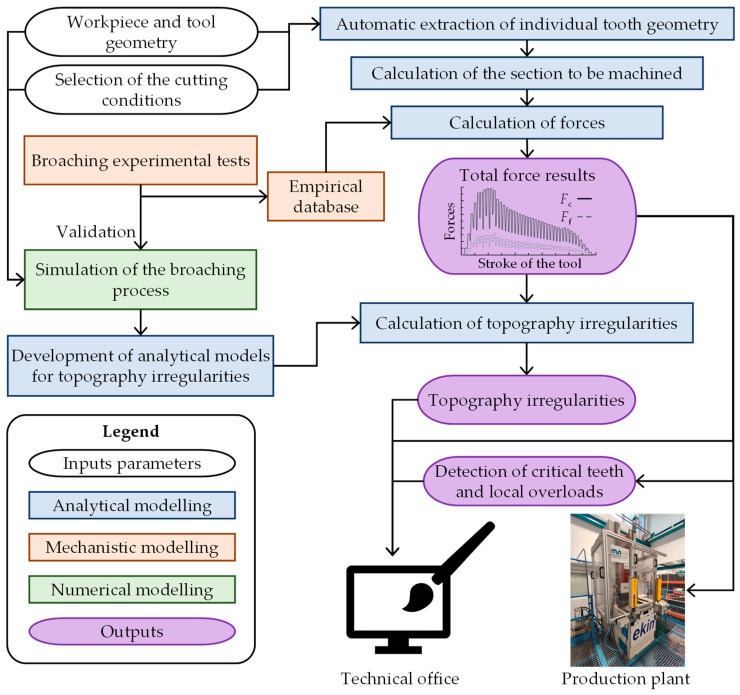
Flow chart of the digital twin to predict forces, local loads, and surface topography irregularities.

The digital twin was implemented with self-developed software programmed in Matlab (R2023B) that incorporates a hybrid model where empirical, numerical, and analytical surrogate models are combined. The general operation of the digital twin is presented in the flow chart in Figure 1 and is based on the following steps:

1. Import the tool and workpiece geometry in STL format and define the necessary input parameters entered by the user: tool and workpiece materials, rake and relief angles, cutting edge radius, coating, pitch, number of teeth, cutting speed, skew angle, and lubrication. Most of these data are used to extract specific cutting forces from the database.
2. Automatically determine the geometry of each tooth using the routine triangle/triangle intersection checking algorithm [34].
3. Calculate the forces per tooth by analytical modelling using the database of specific forces and material section to be machined. In addition, it detects the areas of the cutting edge that withstand a higher local load.
4. Calculate surface topography irregularities taking into account the forces obtained. Analytical equations are used to determine the irregularities based on previously calculated forces. These equations are obtained by performing FEM simulations and experimental broaching tests.

**Figure 2:**
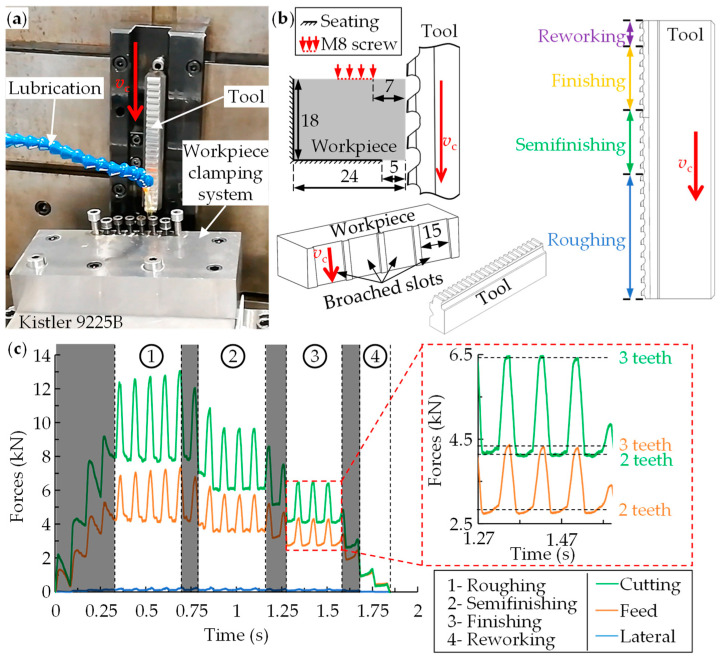
(**a**) Experimental setup of the broaching experimental tests, (**b**) clamping system of the workpiece and geometry of the tool and workpiece, and (**c**) example of forces in Inconel 718.

Hence, to calculate the machining forces and local loads, a specific forces empirical database is required. For this purpose, experimental broaching tests were carried out using an EKIN RAS 10 × 160 × 320 hydraulic broaching machine, equipped with a Kistler 9255B dynamometer (response threshold < 0.01 N) (Kistler Group, Winterthur, Switzerland)to measure forces during the test (see Figure 2a). A rectangular 18 mm thick workpiece and a tool with 8 mm pitch was used, which ensured that 2–3 teeth were in contact at the same time. Each tool was made up of 22 teeth which were divided into roughing (10 teeth), semifinishing (5 teeth), finishing (5 teeth), and reworking (2 teeth). The number of teeth in contact and the tool structure were selected to closely replicate industrial conditions. In industry, multiple teeth are always engaged, and tools typically include roughing, finishing, and reworking teeth, with semifinishing teeth added as needed based on component requirements [1,3,5,6,7]. The workpieces were obtained by wire electrical discharge machining to ensure parallelism between the faces of the block. Figure 2b shows the geometry of the tool and workpiece, as well as a schematic of the workpiece clamping system.

The Design of Experiments (DOE) statistical technique with a central point was used to analyse in a structured way the influence of the input parameters on the output variables and to develop a more robust database. Specifically, the tests analysed the influence of the rake angle, rise per tooth, cutting speed, and workpiece material on the specific forces and topography. Additional tests were carried out to analyse the influence of skew angle and lubrication, but no significant effects were observed. Table 1 shows the complete experimental plan. At least three repetitions of each condition were carried out.

Matlab software was used to capture the forces with a sampling frequency of 5000 Hz and a cut-off filter of 300 Hz (see Figure 2c). The shaded areas were discarded for the analysis, as they correspond to the entry of the teeth at the beginning of the test and to the moments where the teeth in contact have different rise per tooth. For the calculation of the specific cutting (*K*_sc_) and feed (*K*_sf_) forces, Equations (1) and (2) were applied, where *F*_c_ is the cutting force, *F*_f_ the feed force, *f* the rise per tooth, *a*_p_ is the width of cut, and *n*_t_ the number of teeth in contact at the moment of analysis.

(1) $$K_{sc}=\frac{F_{c}}{f\cdot a_{p}\cdot n_{t}}$$

(2) $$K_{sf}=\frac{F_{f}}{f\cdot a_{p}\cdot n_{t}}$$

The database obtained from these tests has specific force values that depend on the uncut chip thickness (rise per tooth), cutting speed, and rake angle for six materials: AISI 1045, Ti-6Al-4V, Inconel 718, 100Cr6, 42CrMo4, and Udimet 720 Li. In total, the digital twin incorporates specific force results from more than 2500 experimental tests.

**Figure 3:**
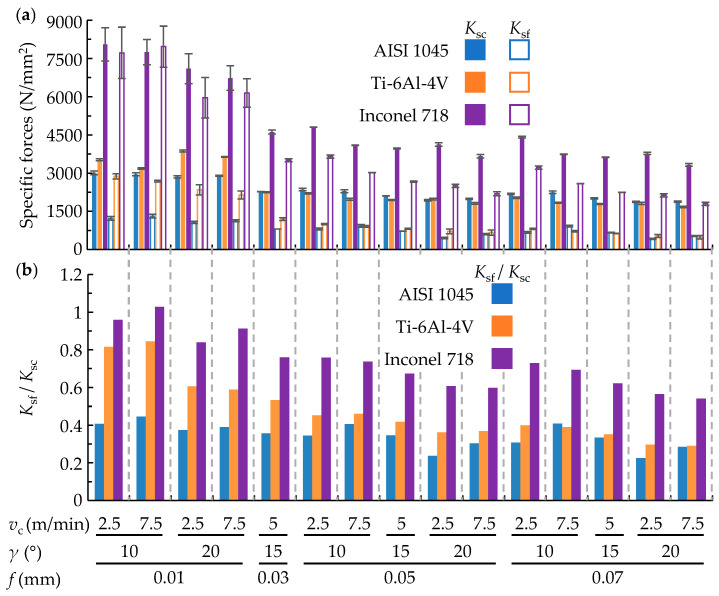
Experimental (**a**) specific force and (**b**) resultant force directionality results.

As an example, Figure 3a shows the values of experimental specific forces of three materials: Inconel 718, Ti-6Al-4V, and AISI 1045 as a function of cutting speed (*v*_c_), rise per tooth (*f*), and rake angle (*γ*). The increase in *f*, *v*_c_, and *γ* produce the reduction in the specific forces in all three materials. *f* has a more significant effect at low feeds (going from 0.01 mm to 0.05 mm) primarily due to the increased ploughing effect, which occurs because of the similarity between the cutting edge radius (5 µm) and the lowest feed. It should also be noted that the specific forces of Ti-6Al-4V and AISI 1045 are very similar, whereas they are 2–3 times higher in Inconel 718. As for the orientation of the resultant force (*K*_sf_/*K_s_*_c_) presented in Figure 3b, significant variations are seen between materials (Inconel 718 ≈ 0.6–1; Ti-6Al-4V ≈ 0.36–0.84; AISI 1045 ≈ 0.25–0.45) and cutting conditions (decreases with increasing *f* and *γ*). The higher the *K*_sf_/*K*_sc_, the more the resultant force is directed towards the workpiece, which significantly affects the topography errors that are directly linked to the surface topography irregularities found.

**Figure 4:**
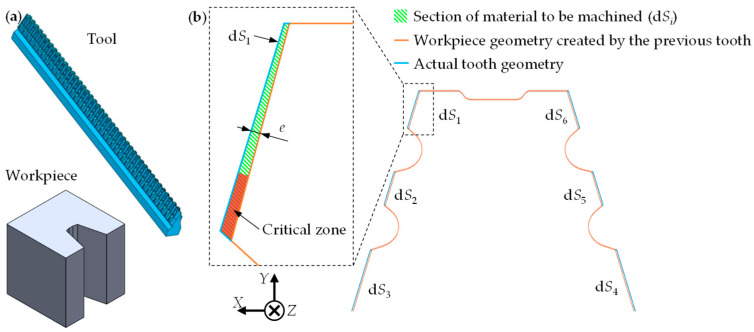
Example of (**a**) a 3D model of a fir-tree workpiece and tool and (**b**) the material section to be machined between two teeth.

Based on the specific forces and using analytical models, the force supported by each tooth and the local load is calculated. First, the section of material to be machined for each tooth (Δ*S_j_*) is calculated based on the 3D STL model of the workpiece and tool (see example in Figure 4a). This section is obtained by the sum of each of the local sections of material to be machined (d*S_i_*), automatically extracted by the digital twin (see Figure 4b).

The model calculates the d*S_i_* incrementally across the cutting edge, taking into account the variations in the local uncut chip thickness (*e*), allowing the identification of the cutting edge zones that withstand higher local loads. Figure 4b shows an example with varying *e* along the cutting edge where the critical zone is the one with the highest *e* (marked in red).

After calculating the section to be machined per tooth, the specific forces are determined with the information in the database, considering the workpiece material and the cutting conditions selected.

Finally, the cutting forces (*F*_c_) and feed (*F*_f_) are calculated at each instant, taking into account the number of teeth machining simultaneously, the specific forces and the section to be machined per tooth (see Equations (3)–(5)).

(3) $$F_{c}=\sum_{j=l-p_{l}}^{l}\;\sum_{i}^{n}\;K_{sc}dS_{ij}$$

(4) $$F_{f}=\sum_{j=l-p_{l}}^{l}\;\sum_{i}^{n}\;K_{sf}dS_{ij}$$

(5) $$\sum_{i}^{n}\;dS_{ij}=\Delta S_{j}=S_{j}-S_{j-1}$$

where *n* is the number of cutting edges in contact on each tooth (Figure 4b shows an example with six cutting edges), *l* is the last tooth in contact with the workpiece at a given instant, *p*_l_ is the number of teeth in contact simultaneously, *j* is the number of teeth, d*S_ij_* is the section of material to be machined of all teeth cutting simultaneously, and Δ*S_j_* is the difference in the section of material to be machined between a given tooth (*S_j_*) and the previous one (*S_j_*_−1_).

**Figure 5:**
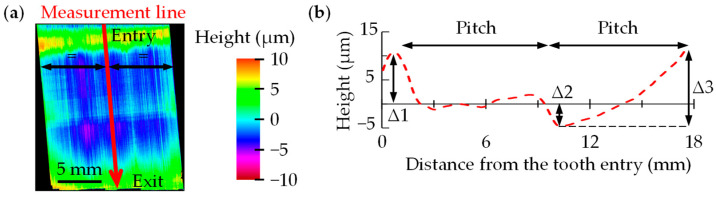
(**a**) Example of the topography of a broached surface of Inconel 718 including (**b**) 2D representation of the measured topography irregularities.

After calculating the forces, the digital twin can quickly predict the surface topography irregularities using analytical equations. These equations were developed by combining experimental tests with FEM simulations. To develop and validate the analytical equations that predict the topography, the experimental broaching tests carried out for the development of the database were used. The topography of all broached groove surfaces (≈15 × 18 mm) was measured using an Alicona IFG4 optical profilometer (Alicona Imaging GmbH, Raaba, Austria) with 20× magnification and vertical and lateral resolutions of 60 nm and 4 μm, respectively. Each surface was composed of more than 100 million points. An example of the characterisation of the topography of a groove can be seen in Figure 5a. The topography profiles were extracted in the centre of the broached slot along the entire machined length (see Figure 5a,b). Figure 5b shows an example of the topography irregularities measured on Inconel 718, where three key features are identified: Δ1 at the entrance of the tooth, Δ3 at the exit, and Δ2 separated from Δ1 and Δ3 at a distance equal to the tool pitch.

Orthogonal cutting (2D) FEM simulations were carried out using AdvantEdge V7.9 finite element software to establish the relationship between cutting forces and surface topography irregularities generated during broaching. The orthogonal cutting configuration was chosen as it is geometrically the most similar to broaching (low rise per tooth compared to the depth of cut) and provides robust results, with reduced computational time, compared to 3D FEM simulations. Details of the models are given in the previously published work in [8].

Combining FEM simulations with experimental topography results confirms that the final surface deformations (Δ1–3, see Figure 5b) are proportional to the applied force and inversely proportional to the Young’s modulus of the tool material (*E*_tool_) or the workpiece (*E*_workpiece_). Applying the theory of elasticity, the displacement of a point *i* of an elastically deformed part depends on the forces and the stiffness (or its inverse, the flexibility, *f_i_*). By linear superposition, this displacement can be obtained from Equation (6). The constants of flexibility in the cutting direction *f*_ic_ and in the feed direction *f_if_* depend on the material, geometry, and clamping conditions. Therefore, if Young’s modulus (*E*_tool_ or *E*_workpiece_, respectively) is applied, the equation can be rewritten as Equation (7). It can be simplified by including the constants *C_if_* and *C_ic_* (see Equation (8)). Subsequently, the equation can be nondimensionalised by dividing by *F*_c_ to express the deformation (Δ*i*) proportional to *F*_c_ and dependent on the direction of the resultant force (*F_f_*/*F_c_*) (see Equation (9)) [8].

(6) $$\Delta i=f_{if}F_{f}+f_{ic}F_{c}$$

(7) $$\Delta iE_{i}=E_{i}f_{i}F_{f}+E_{i}f_{ic}F_{c}$$

(8) $$\Delta iE_{i}=C_{if}F_{f}+C_{ic}F_{c},\;\mathrm{where}\;C_{if}=E_{i}\cdot f_{if}\;y\;C_{ic}=E_{i}\cdot f_{ic}$$

(9) $$\frac{\Delta iE_{i}}{F_{c}}=C_{if}\left(\frac{F_{f}}{F_{c}}\right)+C_{ic}$$

Finally, the experimental data were fitted to Equation (8), where, guided by the simulations, *E_i_* was chosen as *E*_workpiece_ for Δ2 and Δ3, and *E*_tool_ for Δ1, obtaining Equations (10)–(12) [8]. These analytical equations are the ones that were implemented in the digital twin to predict the topography irregularities based on the calculated forces.

(10) $$\Delta 1E_{\mathrm{tool}}=23.1F_{f}-4.6F_{c}$$

(11) $$\Delta 2E_{\mathrm{workpiece}}=-7.9F_{f}+1.5F_{c}$$

(12) $$\Delta 3E_{\mathrm{workpiece}}=31.2F_{f}-5.0F_{c}$$

### 2.2. Validation

**Figure 6:**
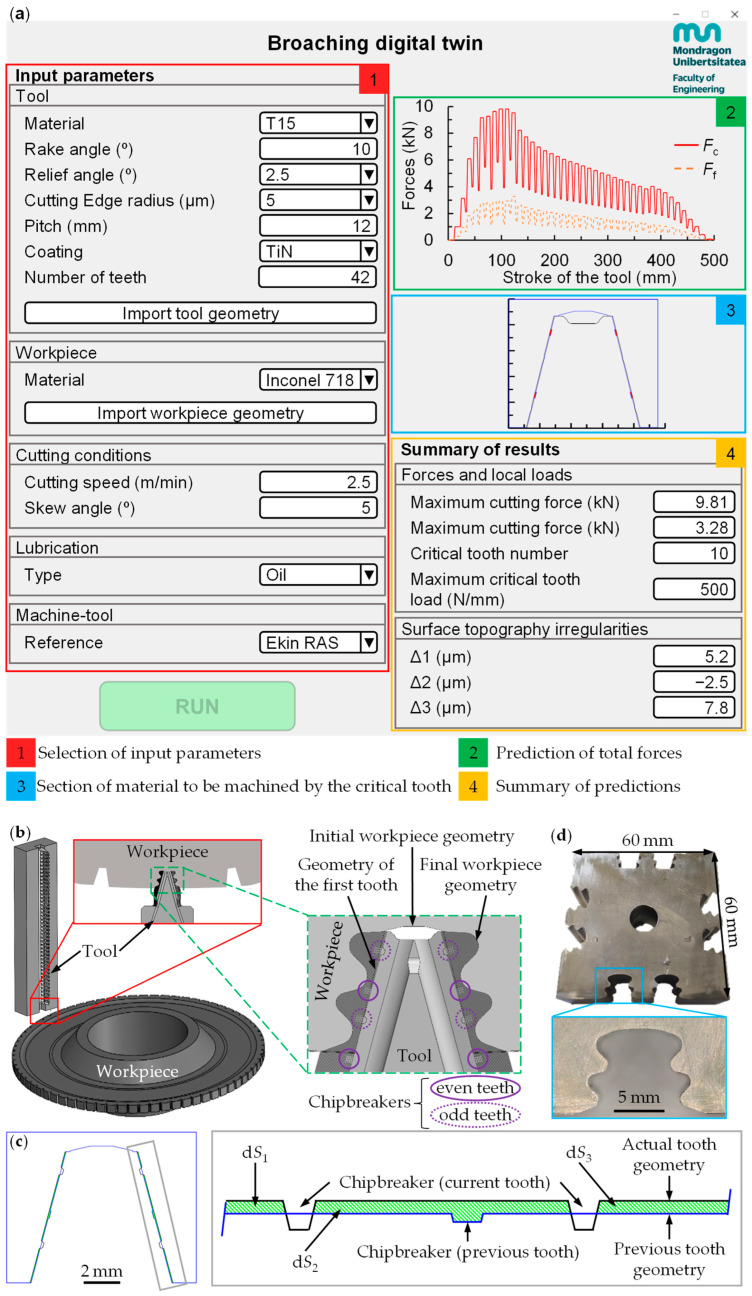
(**a**) Digital twin user interface displaying validation test results, (**b**) 3D model of the workpiece and tool used for validation, (**c**) schematic of a chipbreaker, and (**d**) final workpiece after validation tests.

The digital twin (see user interface in Figure 6a) was validated with one of the most critical broaching processes: the manufacture of the fir-tree feature in turbine discs for aeroengines (see Figure 6b). The fir-tree is usually considered the most critical zone of the turbine disc from the perspective of static and fatigue approaches, as it must withstand high mechanical stresses (induced by centrifugal forces) and thermal stresses [35].

The same machine used in the previous experimental broaching tests was employed (see Figure 2a). A T15 High-Speed Steel tool from a set of broaching tools was selected, with the initial workpiece geometry pre-machined by other tools (see Figure 6b). The broaching tool had two chipbreakers per side on its first nine teeth, whose position varied between the odd and even teeth. On the even teeth, two chipbreakers extend outside the workpiece and had no effect. Chipbreakers enable the formation of discontinuous chips in the width of cut direction and machining sections (d*S_i_*), facilitating chip evacuation. Figure 6c illustrates an example where the chipbreaker divides the material to be machined into three sections (d*S*_1–3_).

Inconel 718 is the most commonly used material in turbine discs [20]. To ensure industrially relevant results, forged and heat-treated (solution treated at 980 °C followed by a double stage ageing at 720 °C and 620 °C) Inconel 718 (average grain size of ASTM 9 and 45 HRC) was chosen as workpiece material. The chemical composition of the workpiece and tool materials are presented in Table 2.

An experimental plan was carried out in which the cutting speed (2.5 and 5 m/min) and the skew angle (0 and 5°) were varied. Two repetitions of each condition were carried out, performing a total of 8 tests (see Figure 6d). All tests were carried out in wet conditions using Cut Max 600 cutting oil at a flow rate of 1.5 L/min.

The output parameters validated included the section of material to be machined for each tooth, the total forces, the local loads, and the topography irregularities. The section of material to be machined obtained from the digital twin was compared to the results obtained manually using AutoCAD design software(AutoCAD 2023). For the forces, the same signal processing methodology as for the other experimental broaching test was followed. Surface topography irregularities were measured by combining the Alicona IFG4 (20× magnification and vertical and lateral resolutions of 60 nm and 4 μm, respectively) with the Mitutoyo Surftest SJ-500 contact profilometer (Mitutoyo Corporation, Kawasaki, Japan), as the latter is more commonly used in the aeronautic industry. In the contact profilometer, the 2 µm radius tip was used, and values were measured every 2 µm across the entire broached surface (≈20 mm), resulting in 10,000 points per profile.

As highlighted in the literature review, surface topography irregularities vary across the broaching direction [7,8] and this could also affect the distribution of other surface-sensitive parameters such as residual stresses. Furthermore, the latter can significantly contribute to the fatigue performance of the manufactured part. Thus, complementarily, surface residual stresses were measured across the bottom surface of the fir-tree to verify if they remain constant or are affected by broaching forces and surface topography irregularities. Surface residual stresses were measured by the X-ray diffraction technique employing Proto iXRD equipment (Proto Manufacturing Ltd., LaSalle, Canada). A round collimator of 1 mm was used to measure surface residual stresses in the broaching direction, in a total of 18 points spaced by 1 mm across the bottom surface of all the manufactured fir-trees. MnKα radiation was used (λ = 2.103 Å), employing a 25 kV voltage and 5 mA current. The beam was tilted at eleven positions from *ψ* = −41° to 41° to apply the sin^2^*ψ* method. At each tilting angle, 10 exposures of 2 s were performed to acquire data on the (311) diffraction peak. PROTO XrdWin software (PROTO XrdWin 2.0) was used to analyse experimental data. The diffraction elastic constants used in the measurements were −*S*_1_ = 1.61 × 10^−6^ (MPa^−1^), ½*S*_2_ = 7.14 × 10^−6^ (MPa^−1^).

## 3. Results and Discussion

This section presents and discusses the validation results from an industrial case study in the aeronautical sector, where the fir-tree feature was broached in Inconel 718. The results from both the digital twin and experimental analyses are presented, focusing on the output variables of total forces, local loads, surface topography, and surface residual stresses.

### 3.1. Forces and Local Loads

**Figure 7:**
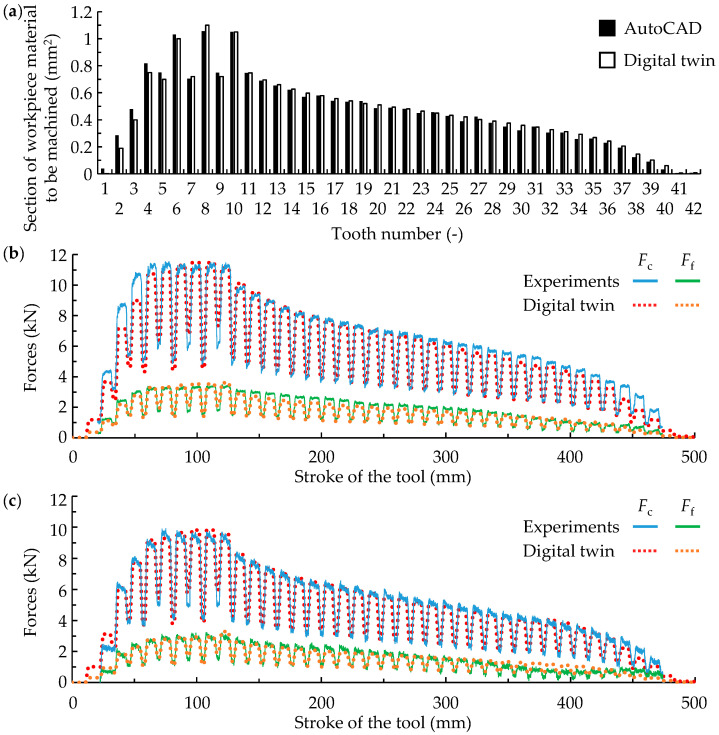
Results of validation tests carried out in Inconel 718: (**a**) section of material to be machined per tooth and (**b**,**c**) forces for cutting speeds of (**b**) 2.5 m/min and (**c**) 5 m/min.

Figure 7a compares the section of material to be machined for each tooth obtained with the digital twin and AutoCAD. As can be seen, the digital twin accurately predicts the section, with an average error of less than 6%.

Subsequently, the machining forces were analysed. The variation in the experimental forces between repetitions was less than 5%. The skew angle, which promotes smoother cut entry, only affected the lateral force, with minimal impact on cutting and feed forces (less than 10% relative to the cutting force). The main differences appeared when the cutting speed was changed. Figure 7b,c show a representative example for each cutting speed. As in the results for the specific forces (see Figure 3), the forces obtained when machining the fir-tree increased as the cutting speed was reduced, as also observed in the literature [36].

The force predictions from the model showed a high degree of accuracy in replicating both the shape and magnitude of the experimental forces for the two cutting speeds. The average relative error considering all tests was less than 10%. Notably, for the teeth under the highest loads (teeth 5 to 10), the error was less than 5%.

The digital twin also enabled the identification of critical teeth and cutting edge sections prone to breakage based on local load calculation. According to the results in Figure 7a, teeth 6, 8, and 10 are most susceptible to breakage due to the larger material sections they must machine. This occurs because the even teeth withstand greater loads than the odd ones, as they have to machine the four chipbreaker areas that the odd ones have not machined, while the odd ones only have to machine two (see Figure 6b).

**Figure 8:**
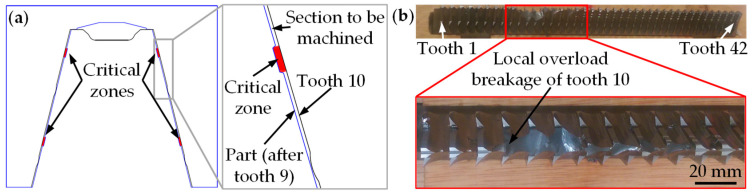
(**a**) Identification of the most critical zones by the digital twin of the validation tests based on local load calculations, and (**b**) breakage of tooth 10 due to local overload.

Among the teeth with the highest forces, the digital twin identified tooth 10 as the most prone to breakage due to the higher local load (see Figure 6a and Figure 8a). This tooth is the first without a chipbreaker and begins forming the fir-tree lobes. As a result, it not only machines its designated nominal rise per tooth but also the section left unmachined by the chipbreaker of tooth 9. The digital twin therefore detected in these zones that the undeformed chip thickness doubles compared to the average rise per tooth of the whole broaching tool, increasing the local load and making this area the most vulnerable to breakage.

Interestingly, once the eight validation tests were completed, the broaching tests were continued under the same machining conditions. After a series of tests in which flank wear did not exceed 0.1 mm, tooth 10 failed, leading to the failure of subsequent teeth 11–13 (see Figure 8b). This confirms that the digital twin is able to correctly predict which tooth is most prone to breakage.

In the future, analytical or FEM surrogate models could be integrated into the digital twin to calculate the stresses along the cutting edge of each tooth, not only identifying the location of the highest local loads, but also determining whether the tooth will break.

### 3.2. Surface Topography

**Figure 9:**
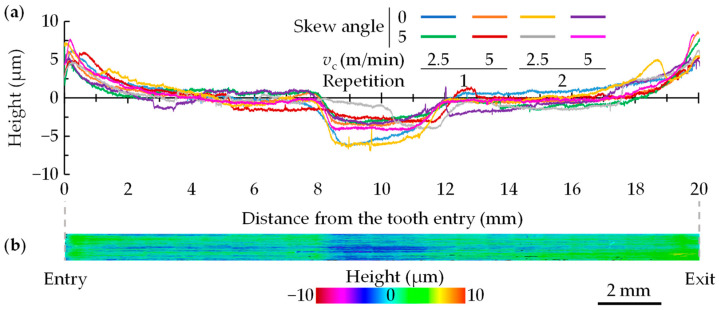
(**a**) Experimental surface topography results of the validation tests and (**b**) the surface from Alicona IFG4 for the 2nd repetition of *v*_c_ = 5 m/min and a skew angle of 5°.

Figure 9a shows all the results of the topography measurements made on the fir-trees including in Figure 9b an image of the surface obtained with Alicona IFG4. As can be seen, the same trend in topography irregularities is observed in all cases, with Δ1 and Δ3 being positive protrusions and Δ2 negative. Moreover, the periodicity coincides with the tool pitch (12 mm). Similar results were observed in the experimental tests with rectangular tools (see Figure 5) [7,8].

Since the topography is force-dependent, and no significant differences in forces were observed between the tested conditions, no clear influences of the cutting speed on the topography were seen either. Across all tests, the mean values were 5.6 ± 0.5 µm for Δ1, −3.5 ± 0.4 µm for Δ2, and 7.4 ± 1 µm for Δ3. The digital twin predicted values of 5.2 µm for Δ1, −2.5 µm for Δ2, and 7.8 µm for Δ3 (see Figure 6a), resulting in an average relative error of less than 15% compared to experimental tests. Therefore, the developed digital twin can effectively predict the topography irregularities generated by broaching that could affect the fretting fatigue behaviour of the component.

### 3.3. Surface Residual Stresses

**Figure 10:**
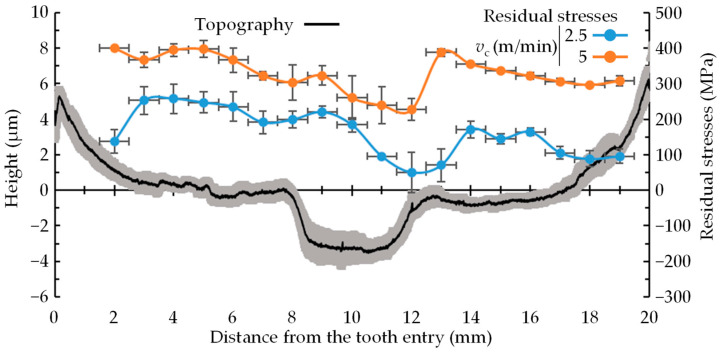
Distribution of surface topography and residual stresses in the broaching direction of the validation tests.

Figure 10 compares the distribution of surface residual stresses across the bottom of the fir-tree for all the tested conditions. No difference in residual stresses was observed due to the skew angle. Therefore, the figure presents the average and standard deviation of all measurements obtained from surfaces machined at cutting speeds of 2.5 and 5 m/min. In addition, using a 1 mm diameter round collimator captures data across that entire area. As a result, a positioning uncertainty of ±0.5 mm along the broached surface was applied.

It should be noted that the highest residual stresses were below 30% of the yield stress of the material, showing that the applied conditions were gentle. On average 200 MPa higher tensile residual stresses were generated when broaching at 2.5 m/min than at 5 m/min. It is widely accepted that cutting-induced residual stresses depend on two major effects [37]: (i) tensile residual stresses are induced near the surface due to the thermal effect, which is even more relevant in materials with low thermal conductivity such as Inconel 718, and (ii) more compressive residual stresses are generated due to the mechanical action caused by cutting forces. The results shown in Figure 7b,c are consistent with these mechanisms. Broaching at 5 m/min increased the heat generated by rubbing/cutting the material, leading to more tensile residual stresses. Additionally, cutting forces were slightly lower when broaching at 5 m/min and consequently the mechanical effect that causes compressive residual stresses was lower too.

Although the magnitude of surface residual stresses changed depending on the broaching conditions, the trends of the distribution along the broached region were the same for all tested conditions (see Figure 10). Surface residual stresses remained significantly constant from entry to the final position, but in the central region of the broached surface, there was a sharp decrease in tensile residual stress magnitude. The change in residual stresses occurs at the same distance as the tool pitch due to the entry and exit effect of the teeth.

This change in the residual stress profile is in good agreement with the previously reported surface topography irregularities (see Figure 8 for all the results and Figure 10 for the average value, with shading representing the standard deviation). The entry and exit of the teeth produce elastic deformations of the tool and workpiece with a frequency equal to the pitch due to the variation in forces. Broaching forces significantly increase when a new tooth reaches the cutting region, leading to an increase in the mechanical effect and consequently generating more compressive residual stresses (see TA 10). Therefore, this analysis confirms that not only surface topography irregularities are induced during broaching but also other parameters of surface integrity (residual stresses) are altered.

Looking ahead, integrating analytical or FEM surrogate models into the digital twin could enable calculations of residual stress variations along the broaching surface. This enhancement would allow the digital twin to provide not only values for total forces, local loads, and surface topography irregularities but also surface residual stresses, offering a comprehensive view of the surface integrity of the broached component.

## 4. Conclusions

This paper presented a digital twin to predict forces, local loads, and topography irregularities in the manufacture of any complex shape geometry by broaching. The digital twin was validated under industrial conditions by performing experimental broaching tests on a fir-tree, which is a critical feature of aircraft turbine discs. This validation test was then complemented with surface residual stress measurements. The main conclusions are presented below.

- The digital twin predicted the force per tooth and would be able to warn the machine operator of unexpected force values due to tool wear or other unforeseen circumstances that could incur tool breakage and risk the surface integrity of the component. It also provided detailed information on the section of material to be machined for each tooth and the location of the most critical tooth area.
- The digital twin facilitated optimisation of the process and tool design to reduce topography irregularities and ensure that geometric and dimensional tolerances are met. As a result, any negative impact on fretting fatigue behaviour that could compromise the functionality of the component was mitigated.
- The predictions were in good agreement with the experimental results of the validation tests, with a relative error in a section of workpiece material to be machined per tooth of less than 6%, in forces of 10%, and in topography irregularities of 15%. The model was also able to identify the most critical zone for local overload.
- Topography irregularities were measured on the fir-tree broaching surface for validation. These variations were governed by the Young’s modulus of both the tool and workpiece, as well as the magnitude and direction of the broaching force. The entry and exit of the teeth during the broaching process caused force fluctuation and, consequently, topography irregularities.
- Residual stresses on the surface of the fir-tree were found to vary not only with cutting speed but also across the broached surface. Specifically, they followed similar patterns to the topography irregularities, with a sharp reduction observed in the central area. Together, these variations in surface integrity indicators could affect the final in-service performance of the component.

The accuracy of the digital twin is considered valid for industrial applications and shows that the specific force database is large enough to correctly predict any of the tooth geometries analysed in this validation case study. Indeed, this same database could be applied to many other complex tool geometries in a wide range of industries. The digital twin could also be extended to any other material by simply entering the specific forces in the database. Hence, the digital twin could be used to modify tool geometry and machining conditions to reduce forces and local loads, or even improve surface integrity by reducing surface topography irregularities via an iterative process.

## Figures and Tables

**Table 1:** Experimental plan for the development of the specific forces database.

**Machine**	Reference	EKIN RAS 10 × 160 × 320
	Actuator	Hydraulic
	Maximum load (kN)	100
**Broaching tool**	Material	T15
	Rake angle, *γ* (°)	10, 15, 20
	Relief angle, *α* (°)	2.5
	Rise per tooth, *f* (mm)	0.01, 0.03, 0.05, 0.07
	Pitch, *P* (mm)	8
	Width, *b* (mm)	15
	Cutting edge radius, *r*_β_ (µm)	5
**Cutting conditions**	Cutting speed, *v*_c_ (m/min)	2.5, 5 and 7.5
	Skew angle (°)	0, 5
**Lubrication**	Type	Dry and wet (Cut Max 600 cutting oil at a flow rate of at 1.5 L/min)
**Workpiece**	Material	AISI 1045, Ti-6Al-4V, Inconel 718, 100Cr6, 42CrMo4, Udimet 720 Li

**Table 2:** Chemical composition (in weight percentage) of nickel-based alloy Inconel 718 and High-Speed Steel T15 (provided by the material supplier).

	Al	C	Co	Cr	Fe	Mo	Ti	V	W	Ni
Inconel 718	0.5	0.004	-	19	18.5	3	0.9	-	-	Bal.
T15	-	1.55	5	4	Bal.	-		5	12	-

## Data Availability

The raw data supporting the conclusions of this article will be made available by the authors on request.
